# The genome sequence of the kissing bug, *Panstrongylus geniculatus *(Latreille, 1811)

**DOI:** 10.12688/wellcomeopenres.23631.1

**Published:** 2025-02-03

**Authors:** Cristian Canizales-Silva, Mateo A. Alvarado-Lopez, Carolina Hernández, Carlos Ospina, Gustavo A. Vallejo, Martin S. Llewellyn, Juan David Ramírez, Wellcome Sanger Institute Tree of Life Management, Samples and Laboratory team, Wellcome Sanger Institute Scientific Operations: Sequencing Operations, Wellcome Sanger Institute Tree of Life Core Informatics team, Tree of Life Core Informatics collective

**Affiliations:** 1 Centro de Investigaciones en Microbiología y Biotecnología de la Universidad del Rosario (CIMBIUR), Universidad del Rosario Facultad de Ciencias Naturales y Matematicas, Bogotá, Bogota, Colombia; 2 Laboratorio de Investigación en Parasitología Tropical, Universidad del Tolima Facultad de Ciencias, Ibagué, Tolima, Colombia; 3 School of Biodiversity, One Health and Veterinary Medicine, University of Glasgow, Glasgow, Scotland, UK; 4 Molecular Microbiology Laboratory, Department of Pathology, Molecular and Cell-Based Medicine, Icahn School of Medicine at Mount Sinai, New York, New York, USA

**Keywords:** Panstrongylus geniculatus, kissing bug, genome sequence, chromosomal, Hemiptera

## Abstract

We present a genome assembly from an individual female *Panstrongylus geniculatus* (kissing bug; Arthropoda; Insecta; Hemiptera; Reduviidae). The assembly contains two haplotypes with total lengths of 1,362.73 megabases and 1,342.40 megabases, respectively. Most of haplotype 1 (97.5%) is scaffolded into 12 chromosomal pseudomolecules. Haplotype 2 is assembled to scaffold level. The mitochondrial genome has also been assembled and is 17.44 kilobases in length.

## Species taxonomy

Eukaryota; Opisthokonta; Metazoa; Eumetazoa; Bilateria; Protostomia; Ecdysozoa; Panarthropoda; Arthropoda; Mandibulata; Pancrustacea; Hexapoda; Insecta; Dicondylia; Pterygota; Neoptera; Paraneoptera; Hemiptera; Prosorrhyncha; Heteroptera; Euheteroptera; Neoheteroptera; Panheteroptera; Cimicomorpha; Reduvioidea; Reduviidae; Triatominae; Panstrongylus; *Panstrongylus geniculatus* (Latreille, 1811) (NCBI:txid156442)

## Background

**Figure 1.**
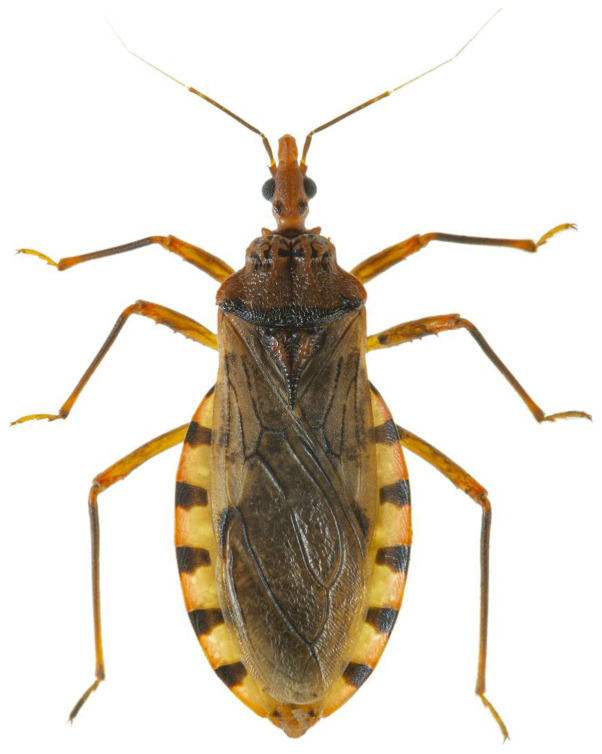
Photograph of *Panstrongylus geniculatus* by SMUCV (not the specimen used for genome sequencing).

We present the reference genome of *Panstrongylus geniculatus* (Latreille, 1811) ( Figure 1), a widely distributed triatomine insect throughout the Americas, excluding Chile, with records from Mexico, Central America, South America, and island nations like Trinidad ( Galvão *et al.*, 2003; Gurgel-Gonçalves *et al.*, 2012; Leite *et al.*, 2007; Tineo-González *et al.*, 2023). This hematophagous insect inhabits rural, urban, and suburban environments across dry, humid, and rainforest regions ( Patterson *et al.*, 2009). Due to its domiciliation potential, *P. geniculatus* is a significant vector of *Trypanosoma cruzi*, the etiological agent of Chagas disease (CD) ( Nakad Bechara *et al.*, 2018; Reyes-Lugo & Rodriguez-Acosta, 2000). This neglected disease affects approximately 6–7 million people primarily in Latin America, with over 10,000 deaths annually ( Silvestrini *et al.*, 2024; World Health Organization, 2022), posing a substantial public health concern.

Adult *P. geniculatus* exhibit sexual dimorphism, with females distinguished by a pointed posterior end and slightly larger body size (22.5–29.5 mm) compared to males (22–28 mm). The insect exhibits a colour pattern ranging from light brown to orange-brown, with irregular black or dark brown patches. The legs vary in colour from orange to dark brown, typically lighter at the joints. A distinctive feature is the connexivum, which alternates between matte black or dark brown and ochre ( Vivas *et al.*, 2021).

Studies on *P. geniculatus* life cycle under laboratory conditions show that it deposits eggs freely on surfaces rather than attaching them to substrates. The eggs initially appear bright white and turn pink after approximately a week. The species undergoes five nymphal stages before reaching adulthood, with development ranging from 149 to 531 days influenced by temperature, humidity, and food availability ( Vivas *et al*., 2021). Despite limited research on *Panstrongylus* species phylogenetics, studies such as those by Caicedo-Garzón *et al*. (2019) have identified that *P. geniculatus* is monophyletic, revealing four genetically supported clades possibly influenced by Andean orogeny.

*P. geniculatus* plays a critical role in the transmission of *T. cruzi*, particularly in recent oral outbreaks of CD across Latin America. It was identified as the primary vector in a significant number of cases, contributing to 7 out of 10 outbreaks in Colombia and 4 out of 5 in Venezuela. This highlights its involvement in approximately 73% of recent oral outbreaks ( López-García & Gilabert, 2023).

Originally considered a sylvatic vector primarily feeding on armadillos, opossums, rodents, and bats ( Patterson *et al.*, 2009), recent studies indicate its adaptation to domestic habitats ( Carrasco *et al.*, 2005; Carrasco *et al.*, 2014; Reyes-Lugo, 2009). Arias-Giraldo *et al*. (2020) extensively documented the varied feeding habits of *P. geniculatus*, with its diet including Artiodactyla, Birds, Canidae, Dasypodidae, Didelphidae, Equidae, Felidae, Procyonidae, Chiroptera, Rodents, Vermilingua, and non-human primates, highlighting its role as a bridge vector between wildlife and human dwellings. This broad host range increases the risk of zoonotic transmission events, facilitating the spread of various discrete typing units (DTUs) of *T. cruzi* (TcI – TcV) during oral outbreaks in Colombia and Venezuela ( Hernández *et al.*, 2016; Velásquez-Ortiz *et al.*, 2022).

The assembly of the complete chromosome-level genome of *P. geniculatus* presented here sets the stage for future research into parasite-vector dynamics and targeted vector control strategies aimed at reducing CD prevalence.

## Genome sequence report

The genome of an adult female *Panstrongylus geniculatus* was sequenced using Pacific Biosciences single-molecule HiFi long reads, generating a total of 47.24 Gb (gigabases) from 5.90 million reads, providing approximately 33-fold coverage. Primary assembly contigs were scaffolded with chromosome conformation Hi-C data, which produced 95.25 Gb from 630.77 million reads. Specimen and sequencing details are provided in Table 1.

**Table 1.** Specimen and sequencing data for *Panstrongylus geniculatus*.

Project information			
**Study title**	Panstrongylus geniculatus (kissing bug)		
**Umbrella BioProject**	PRJEB74974		
**Species**	*Panstrongylus geniculatus*		
**BioSample**	SAMEA114214723		
**NCBI taxonomy ID**	156442		
Specimen information			
**Technology**	**ToLID**	**BioSample accession**	**Organism part**
**PacBio long read sequencing**	ihPanGeni1	SAMEA114214733	whole organism
**Hi-C sequencing**	ihPanGeni1	SAMEA114214733	whole organism
Sequencing information			
**Platform**	**Run accession**	**Read count**	**Base count (Gb)**
**Illumina NovaSeq 6000 (Hi-C)**	ERR12945473	6.31e+08	95.25
**Revio (PacBio)**	ERR12921319	2.25e+06	18.57
**Revio (PacBio)**	ERR12921320	3.66e+06	28.67

**Figure 2.**
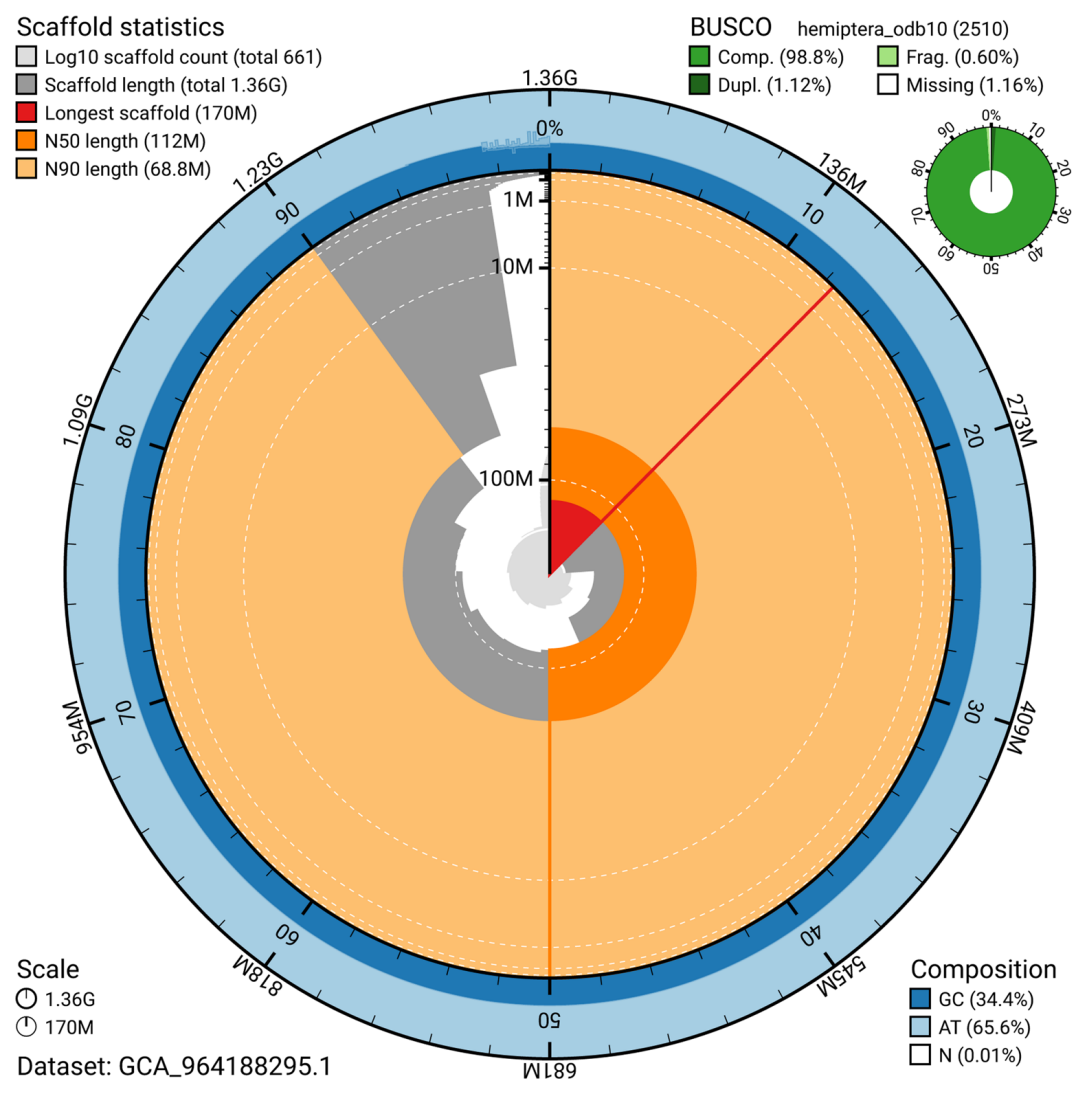
Genome assembly of *Panstrongylus geniculatus*, ihPanGeni1.hap1.1: metrics. The BlobToolKit snail plot provides an overview of assembly metrics and BUSCO gene completeness. The circumference represents the length of the whole genome sequence, and the main plot is divided into 1,000 bins around the circumference. The outermost blue tracks display the distribution of GC, AT, and N percentages across the bins. Scaffolds are arranged clockwise from longest to shortest and are depicted in dark grey. The longest scaffold is indicated by the red arc, and the deeper orange and pale orange arcs represent the N50 and N90 lengths. A light grey spiral at the centre shows the cumulative scaffold count on a logarithmic scale. A summary of complete, fragmented, duplicated, and missing BUSCO genes in the hemiptera_odb10 set is presented at the top right. An interactive version of this figure is available at https://blobtoolkit.genomehubs.org/view/Panstrongylus_geniculatus/dataset/GCA_964188295.1/snail.

**Figure 3.**
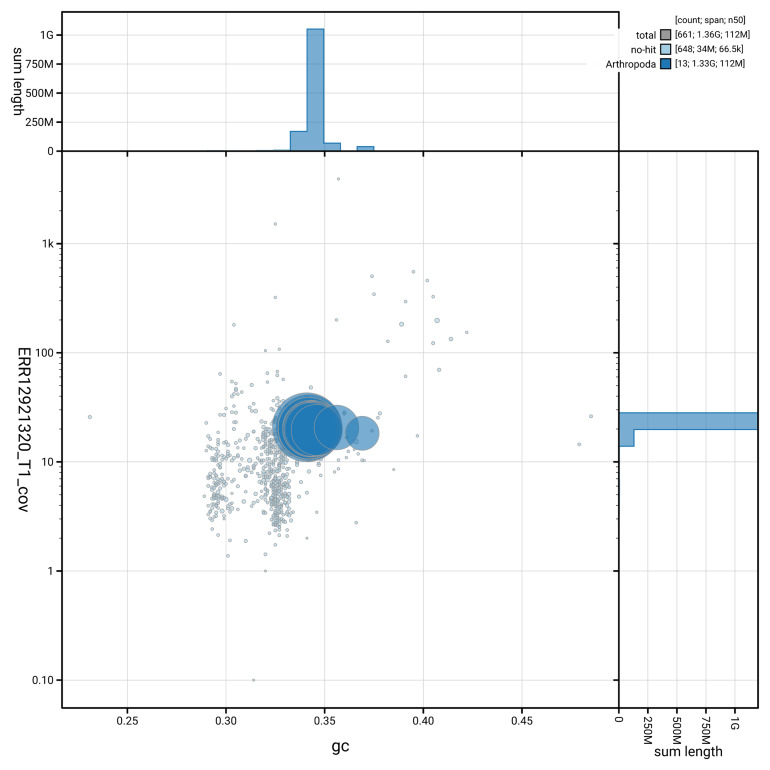
Genome assembly of *Panstrongylus geniculatus*, ihPanGeni1.hap1.1: BlobToolKit GC-coverage plot. Blob plot showing sequence coverage (vertical axis) and GC content (horizontal axis). The circles represent scaffolds, with the size proportional to scaffold length and the colour representing phylum membership. The histograms along the axes display the total length of sequences distributed across different levels of coverage and GC content. An interactive version of this figure is available at https://blobtoolkit.genomehubs.org/view/Panstrongylus_geniculatus/dataset/GCA_964188295.1/blob.

**Figure 4.**
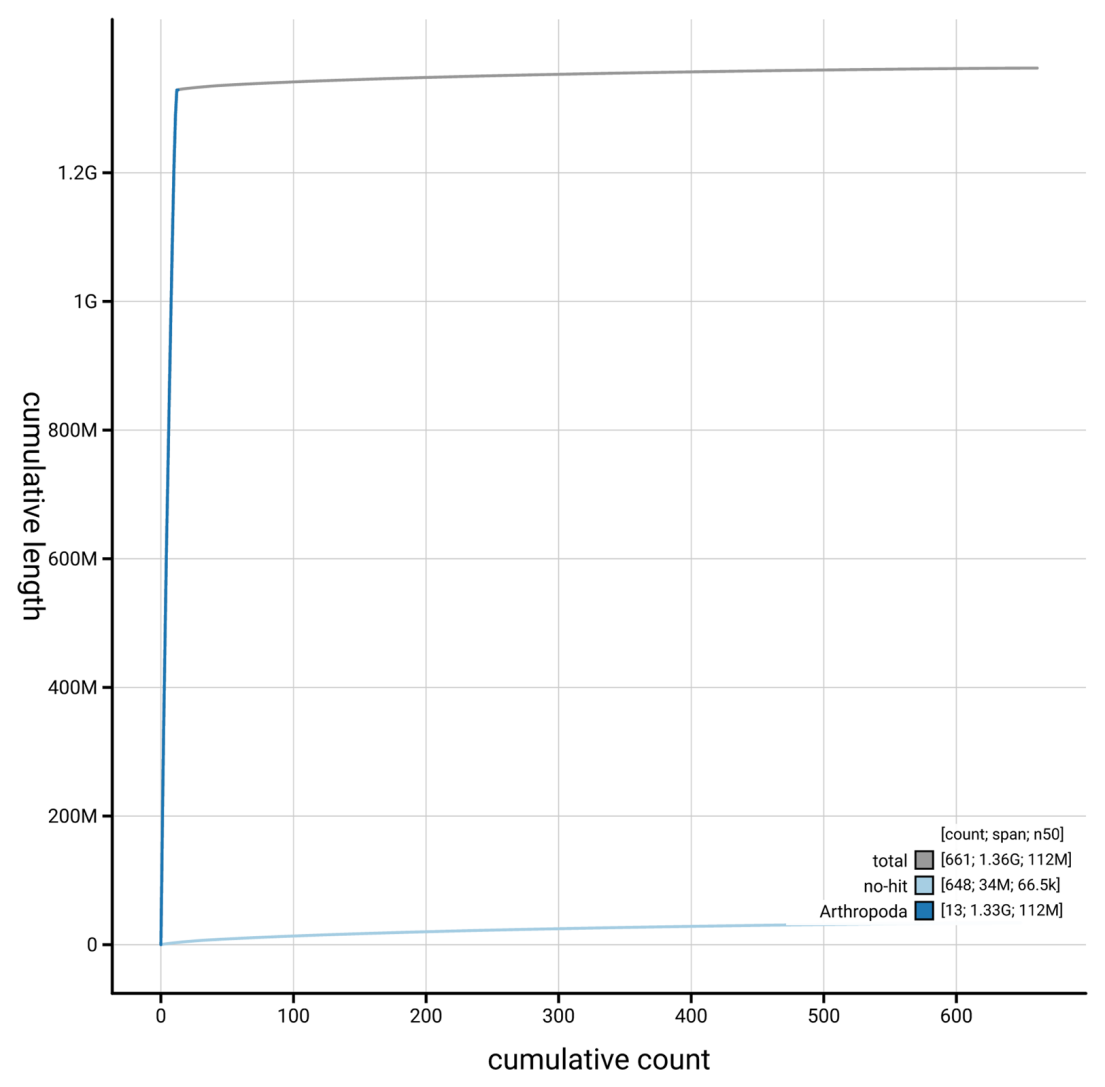
Genome assembly of *Panstrongylus geniculatus* ihPanGeni1.hap1.1: BlobToolKit cumulative sequence plot. The grey line shows cumulative length for all scaffolds. Coloured lines show cumulative lengths of scaffolds assigned to each phylum using the buscogenes taxrule. An interactive version of this figure is available at https://blobtoolkit.genomehubs.org/view/Panstrongylus_geniculatus/dataset/GCA_964188295.1/cumulative.

The two haplotypes were combined for curation. Manual assembly curation corrected 16 missing joins or mis-joins. This reduced the scaffold number by 0.75%. The final haplotype 1 assembly has a total length of 1,362.73 Mb in 660 sequence scaffolds, with 494 gaps, and a scaffold N50 of 112.1 Mb ( Table 2). The snail plot in Figure 2 provides a summary of the assembly statistics, while the distribution of assembly scaffolds on GC proportion and coverage is shown in Figure 3. The cumulative assembly plot in Figure 4 shows curves for subsets of scaffolds assigned to different phyla.

**Table 2.** Genome assembly data for the *Panstrongylus geniculatus* assembly.

Genome assembly	Haplotype 1	Haplotype 2
Assembly name	ihPanGeni1.hap1.1	ihPanGeni1.hap2.1
Assembly accession	GCA_964188295.1	GCA_964188385.1
Assembly level	chromosome	scaffold
Span (Mb)	1,362.73	1,342.40
Number of contigs	1,154	770
Number of scaffolds	660	300
Longest scaffold (Mb)	169.91	None
Assembly metrics *	Haplotype 1	Haplotype 2
Contig N50 length ( *≥ 1 Mb*)	4.76 Mb	4.92 Mb
Scaffold N50 length ( *= chromosome N50*)	112.1 Mb	112.19 Mb
Consensus quality (QV) (≥ 40)	63.8	64.6
*k*-mer completeness	84.36%	84.19%
*k*-mer completeness combined (≥ 95%)	99.19%	
BUSCO ** (S > 90%; D < 5%)	C:98.9%[S:97.7%,D:1.2%], F:0.6%,M:0.5%,n:2,510	C:98.5%[S:97.5%,D:1.0%], F:0.6%,M:0.9%,n:2,510
Percentage of assembly mapped to chromosomes (≥ 90%)	97.5%	-
Sex chromosomes (localised homologous pairs)	X _1_ and X _2_	-
Organelles (one complete allele)	Mitochondrial genome: 17.44 kb	-

* Assembly metric benchmarks are adapted from Rhie *et al.* (2021) and the Earth BioGenome Project Report on Assembly Standards September 2024. ** BUSCO scores based on the hemiptera_odb10 BUSCO set using version 5.4.3. C = complete [S = single copy, D = duplicated], F = fragmented, M = missing, n = number of orthologues in comparison.

**Figure 5.**
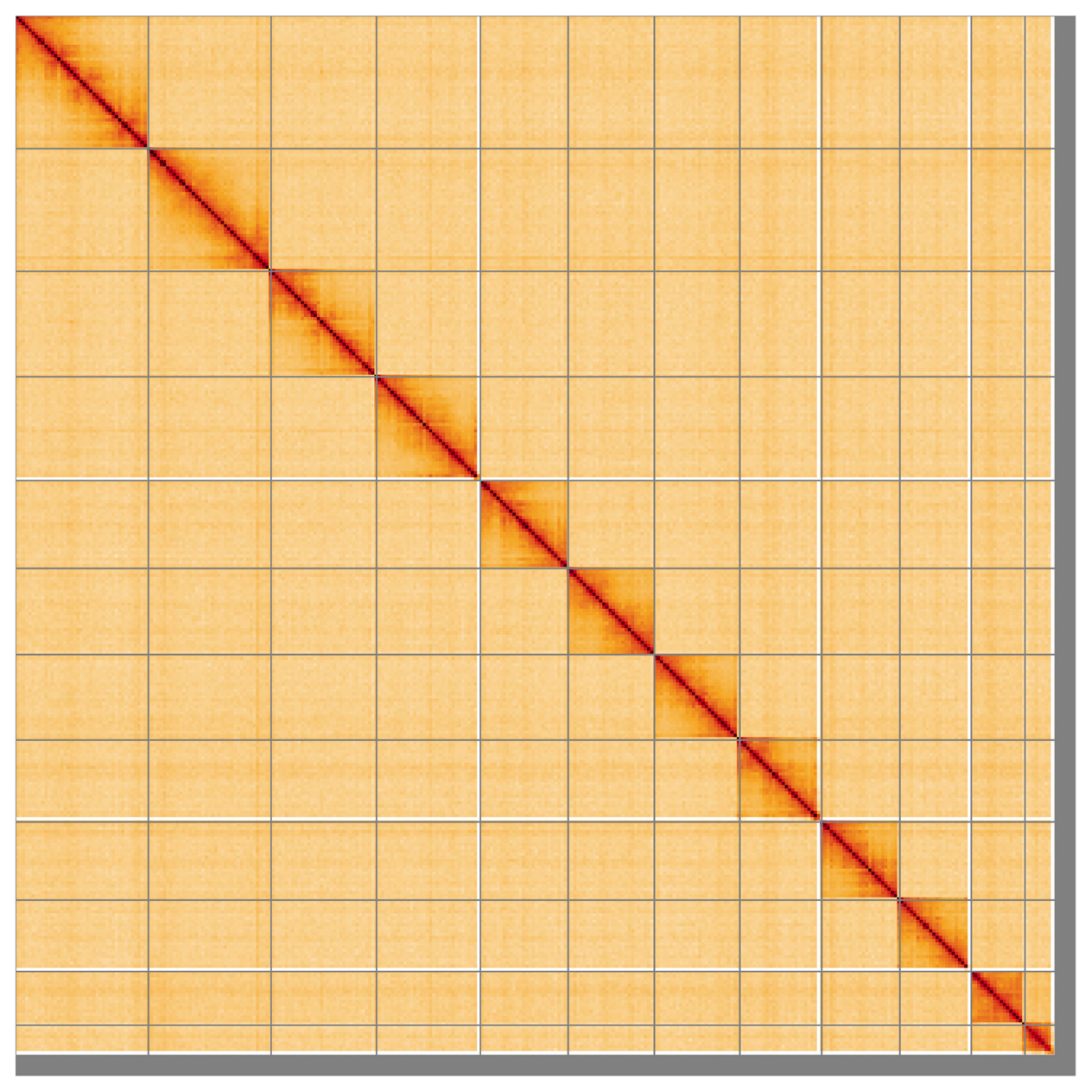
Genome assembly of *Panstrongylus geniculatus* ihPanGeni1.hap1.1: Hi-C contact map of the ihPanGeni1.hap1.1 assembly, visualised using HiGlass. Chromosomes are shown in order of size from left to right and top to bottom. An interactive version of this figure may be viewed at https://genome-note-higlass.tol.sanger.ac.uk/l/?d=Zvb2cinoSg-CrCT9LwsAvA.

Most (97.5%) of the assembly sequence for haplotype 1 was assigned to 12 chromosomal-level scaffolds, representing 10 autosomes and the X _1_ and X _2_ sex chromosomes. Chromosome-scale scaffolds confirmed by the Hi-C data are named in order of size ( Figure 5; Table 3). Chromosomes X _1_ and X _2_ were assigned by synteny to the *Leptopterna dolabrata* assembly (GCA_954871275.1). The karyotype is known from Crossa *et al*. (2002).

**Table 3.** Chromosomal pseudomolecules in the genome assembly of *Panstrongylus geniculatus*, ihPanGeni1.

INSDC accession	Name	Length (Mb)	GC%
OZ076906.1	1	169.91	34
OZ076907.1	2	156.72	34
OZ076908.1	3	134.74	34
OZ076909.1	4	132.61	34
OZ076910.1	5	112.1	34.5
OZ076911.1	6	110.17	34.5
OZ076912.1	7	109.39	34.5
OZ076913.1	8	104.45	34.5
OZ076914.1	9	99.97	34.5
OZ076915.1	10	91.08	34.5
OZ076918.1	MT	0.02	32.5
OZ076916.1	X1	68.81	35.5
OZ076917.1	X2	38.78	37

The mitochondrial genome was also assembled and is included both as a contig within the multifasta file of the genome submission and as a standalone record in GenBank.

### Assembly quality metrics

The estimated Quality Value (QV) and *k*-mer completeness metrics, along with BUSCO completeness scores, were calculated for each haplotype and the combined assembly. The QV reflects the base-level accuracy of the assembly, while *k*-mer completeness indicates the proportion of expected *k*-mers identified in the assembly. BUSCO scores provide a measure of completeness based on benchmarking universal single-copy orthologues.

For haplotype 1, the assembly has an estimated QV of 63.8 and *k*-mer completeness of 84.36%. BUSCO v5.4.3 analysis using the hemiptera_odb10 reference set (n = 2,510) shows a completeness of 98.9% (single = 97.7%, duplicated = 1.2%).

For haplotype 2, the assembly has an estimated QV of 64.6 and *k*-mer completeness of 84.19%. BUSCO v5.4.3 analysis shows a completeness of 98.5% (single = 97.5%, duplicated = 1.0%).

When the two haplotypes are combined, the assembly achieves an estimated QV of 64.2 and *k*-mer completeness of 99.19%.

## Methods

### Sample acquisition

An adult female specimen of *Panstrongylus geniculatus* (specimen ID SAN20001677, ToLID ihPanGeni1) was collected from a laboratory colony maintained by the Universidad del Tolima in Ibague, Colombia by Gustavo Vallejo. The colony was originally established using individuals collected in the municipality of Molagavita, Santander (Colombia) in 2011. They were then reared under controlled conditions in the insectary, with temperatures maintained in the range 24–26°C, relative humidity of 60–70%, and a 12:12 hour photoperiod. The insects were fed on chickens ( *Gallus gallus*). The selected specimens were further identified and subsequently transferred to the Centro de Investigaciones en Microbiología y Biotecnología de la Universidad del Rosario (CIMBIUR) in Bogotá, Colombia, for further processing.

These specimens were then prepared for export as part of the Wellcome Sanger Institute’s Tree of Life (ToL) project. To facilitate the export to the Wellcome Sanger Institute, the required permits were obtained, including an export permit from Colombia’s National Environmental Licensing Authority (ANLA), granted under authorization number 3258 on May 16, 2023. Additionally, the UK Animal and Plant Health Agency issued the corresponding import permit (ITIMP23.0761). All export and import procedures adhered to the requirements of the Darwin Tree of Life project, under agreement number 255420.

### Nucleic acid extraction

The workflow for high molecular weight (HMW) DNA extraction at the Wellcome Sanger Institute (WSI) Tree of Life Core Laboratory includes a sequence of core procedures: sample preparation and homogenisation, DNA extraction, fragmentation and purification. Detailed protocols are available on protocols.io ( Denton *et al.*, 2023b).

The ihPanGeni1 sample was prepared for DNA extraction by weighing and dissecting it on dry ice ( Jay *et al.*, 2023). Tissue from the whole organism was homogenised using a PowerMasher II tissue disruptor ( Denton *et al.*, 2023a). 

HMW DNA was extracted using the Automated MagAttract v2 protocol ( Oatley *et al.*, 2023a). DNA was sheared into an average fragment size of 12–20 kb in a Megaruptor 3 system ( Bates *et al.*, 2023). Sheared DNA was purified by solid-phase reversible immobilisation, using AMPure PB beads to eliminate shorter fragments and concentrate the DNA ( Oatley *et al.*, 2023b). The concentration of the sheared and purified DNA was assessed using a Nanodrop spectrophotometer and Qubit Fluorometer using the Qubit dsDNA High Sensitivity Assay kit. Fragment size distribution was evaluated by running the sample on the FemtoPulse system.

### Hi-C sample preparation

Tissue from the whole organism of the ihPanGeni1 sample was processed at the WSI Scientific Operations core, using the Arima-HiC v2 kit. In brief, 20–50mg of frozen tissue (stored at –80 °C) was fixed, and the DNA crosslinked using a TC buffer with 22% formaldehyde concentration. After crosslinking the tissue was homogenised using the Diagnocine Power Masher-II and BioMasher-II tubes and pestles. Following the Arima-HiC v2 kit manufacturer's instructions, crosslinked DNA was digested using a restriction enzyme master mix. The 5’-overhangs were filled in and labelled with biotinylated nucleotides and proximally ligated. An overnight incubation was carried out for enzymes to digest remaining proteins and for crosslinks to reverse. A clean up was performed with SPRIselect beads prior to library preparation. Additionally, biotinylation percentage was estimated using the Qubit Fluorometer v4.0 (Thermo Fisher Scientific) and Qubit HS Assay Kit and Arima-HiC v2 QC beads.

### Library preparation and sequencing

Library preparation and sequencing were performed at the WSI Scientific Operations core.

***PacBio HiFi sequencing***

At the minimum, samples were required to have an average fragment size exceeding 8 kb and a total mass over 400 ng to proceed to the low input SMRTbell Prep Kit 3.0 protocol (Pacific Biosciences, California, USA), depending on genome size and sequencing depth required. Libraries were prepared using the SMRTbell Prep Kit 3.0 (Pacific Biosciences, California, USA) as per the manufacturer's instructions. The kit includes the reagents required for end repair/A-tailing, adapter ligation, post-ligation SMRTbell bead cleanup, and nuclease treatment. Following the manufacturer’s instructions, size selection and clean up was carried out using diluted AMPure PB beads (Pacific Biosciences, California, USA). DNA concentration was quantified using the Qubit Fluorometer v4.0 (Thermo Fisher Scientific) with Qubit 1X dsDNA HS assay kit and the final library fragment size analysis was carried out using the Agilent Femto Pulse Automated Pulsed Field CE Instrument (Agilent Technologies) and gDNA 55kb BAC analysis kit. Prepared libraries were normalised to 2 nM and 15 μL used for making complexes. For libraries below 2nM all 10uL was used for making complexes. Primers were annealed and polymerases were hybridised to create circularised complexes according to manufacturer’s instructions. The complexes were purified with the 1.2X clean up with SMRTbell beads. The purified complexes were then diluted to the Revio loading concentration, between 200 -300pM, and spiked with a Revio sequencing internal control. Samples were sequenced using the Revio system on Revio 25M SMRT cells (Pacific Biosciences, California, USA). The SMRT link software, a PacBio web-based end-to-end workflow manager, was used to set-up and monitor the run, as well as perform primary and secondary analysis of the data upon completion.

***Hi-C***

For Hi-C library preparation, DNA was fragmented using the Covaris E220 sonicator (Covaris) and size selected using SPRISelect beads to 400 to 600 bp. The DNA was then enriched using the Arima-HiC v2 kit Enrichment beads. Using the NEBNext Ultra II DNA Library Prep Kit (New England Biolabs) for end repair, a-tailing, and adapter ligation. This uses a custom protocol which resembles the standard NEBNext Ultra II DNA Library Prep protocol but where library preparation occurs while DNA is bound to the Enrichment beads. For library amplification, 10–16 PCR cycles were required, determined by the sample biotinylation percentage.

### Genome assembly, curation and evaluation

***Assembly***

The HiFi reads were assembled using Hifiasm ( Cheng *et al.*, 2021; Cheng *et al.*, 2022) in Hi-C phasing mode, resulting in a pair of haplotype-resolved assemblies. The Hi-C reads were mapped to the primary contigs using bwa-mem2 ( Vasimuddin *et al.*, 2019). The contigs were further scaffolded using the provided Hi-C data ( Rao *et al.*, 2014) in YaHS ( Zhou *et al.*, 2023) using the --break option for handling potential misassemblies. The scaffolded assemblies were evaluated using Gfastats ( Formenti *et al.*, 2022), BUSCO ( Manni *et al.*, 2021) and MERQURY.FK ( Rhie *et al.*, 2020).

The mitochondrial genome was assembled using MitoHiFi ( Uliano-Silva *et al.*, 2023), which runs MitoFinder ( Allio *et al.*, 2020) and uses these annotations to select the final mitochondrial contig and to ensure the general quality of the sequence.

***Assembly curation***

The assembly was decontaminated using the Assembly Screen for Cobionts and Contaminants (ASCC) pipeline (article in preparation). Flat files and maps used in curation were generated in TreeVal ( Pointon *et al.*, 2023). Manual curation was primarily conducted using PretextView ( Harry, 2022), with additional insights provided by JBrowse2 ( Diesh *et al.*, 2023) and HiGlass ( Kerpedjiev *et al.*, 2018). Scaffolds were visually inspected and corrected as described by Howe *et al*. (2021). Any identified contamination, missed joins, and mis-joins were corrected, and duplicate sequences were tagged and removed. Sex chromosomes were identified by synteny analysis. The curation process is documented at https://gitlab.com/wtsi-grit/rapid-curation (article in preparation).

***Assembly quality assessment***

The MerquryFK tool ( Rhie *et al.*, 2020), run within a Singularity container ( Kurtzer *et al.*, 2017), was used to evaluate *k*-mer completeness and assembly quality for the curated haplotypes (Hap1/2), using the *k*-mer databases ( *k* = 31) computed prior to genome assembly. The analysis outputs included assembly QV scores and completeness statistics.

To create the HiGlass contact map, Hi-C reads were aligned using bwa-mem2 ( Vasimuddin *et al.*, 2019), and the resulting alignment files were processed with SAMtools ( Danecek *et al.*, 2021). Contact matrices were generated with BEDTools ( Quinlan & Hall, 2010) and the Cooler tool suite ( Abdennur & Mirny, 2020), and the contact map was visualised in HiGlass ( Kerpedjiev *et al.*, 2018).

The assembly was further evaluated using a Nextflow ( Di Tommaso *et al.*, 2017) port of the BlobToolKit pipeline ( Muffato *et al.*, 2024), which builds on the original BlobToolKit implementation ( Challis *et al.*, 2020). This pipeline aligns PacBio reads with SAMtools and minimap2 ( Li, 2018), generating coverage tracks for fixed-size regions. Simultaneously, BUSCO genes were identified from the assembly using the GoaT database ( Challis *et al.*, 2023), and BUSCO completeness scores were calculated ( Manni *et al.*, 2021). For three domain-level BUSCO lineages, DIAMOND blastp ( Buchfink *et al.*, 2021) was used to align BUSCO genes to the UniProt Reference Proteomes database ( Bateman *et al.*, 2023). Additional genome chunks were aligned with DIAMOND blastx and blastn ( Altschul *et al.*, 1990), with sequences without hits aligned to the NT database. The BlobToolKit suite integrated these outputs into a blobdir for visualisation.

The genome evaluation workflows utilised nf-core tooling ( Ewels *et al.*, 2020) and MultiQC ( Ewels *et al.*, 2016). They relied on the Conda package manager, Bioconda ( Grüning *et al.*, 2018), Biocontainers ( da Veiga Leprevost *et al.*, 2017), Docker ( Merkel, 2014), and Singularity ( Kurtzer *et al.*, 2017) containerisation.

Table 4 contains a list of relevant software tool versions and sources.

**Table 4.** Software tools: versions and sources.

Software tool	Version	Source
BEDTools	2.30.0	https://github.com/arq5x/bedtools2
BLAST	2.14.0	ftp://ftp.ncbi.nlm.nih.gov/blast/executables/blast+/
BlobToolKit	4.3.7	https://github.com/blobtoolkit/blobtoolkit
BUSCO	5.4.3 and 5.5.0	https://gitlab.com/ezlab/busco
bwa-mem2	2.2.1	https://github.com/bwa-mem2/bwa-mem2
Cooler	0.8.11	https://github.com/open2c/cooler
DIAMOND	2.1.8	https://github.com/bbuchfink/diamond
fasta_windows	0.2.4	https://github.com/tolkit/fasta_windows
FastK	427104ea91c78c3b8b8b49f1a7d6bbeaa869ba1c	https://github.com/thegenemyers/FASTK
Gfastats	1.3.6	https://github.com/vgl-hub/gfastats
GoaT CLI	0.2.5	https://github.com/genomehubs/goat-cli
Hifiasm	0.19.8-r603	https://github.com/chhylp123/hifiasm
HiGlass	44086069ee7d4d3f6f3f0012569789ec138f42b84aa44357826c0b6753eb28de	https://github.com/higlass/higlass
Merqury.FK	d00d98157618f4e8d1a9190026b19b471055b22e	https://github.com/thegenemyers/MERQURY.FK
Minimap2	2.24-r1122	https://github.com/lh3/minimap2
MitoHiFi	3	https://github.com/marcelauliano/MitoHiFi
MultiQC	1.14, 1.17, and 1.18	https://github.com/MultiQC/MultiQC
NCBI Datasets	15.12.0	https://github.com/ncbi/datasets
Nextflow	23.04.0-5857	https://github.com/nextflow-io/nextflow
PretextView	0.2	https://github.com/sanger-tol/PretextView
purge_dups	1.2.5	https://github.com/dfguan/purge_dups
samtools	1.16.1, 1.17, and 1.18	https://github.com/samtools/samtools
sanger-tol/ascc	-	https://github.com/sanger-tol/ascc
sanger-tol/blobtoolkit	v0.6.0	https://github.com/sanger-tol/blobtoolkit/tree/0.6.0
Seqtk	1.3	https://github.com/lh3/seqtk
Singularity	3.9.0	https://github.com/sylabs/singularity
TreeVal	1.0.0	https://github.com/sanger-tol/treeval
YaHS	1.2a.2	https://github.com/c-zhou/yahs

### Wellcome Sanger Institute – Legal and Governance

The materials that have contributed to this genome note have been supplied by a Tree of Life collaborator.

The Wellcome Sanger Institute employs a process whereby due diligence is carried out proportionate to the nature of the materials themselves, and the circumstances under which they have been/are to be collected and provided for use. The purpose of this is to address and mitigate any potential legal and/or ethical implications of receipt and use of the materials as part of the research project, and to ensure that in doing so we align with best practice wherever possible.

The overarching areas of consideration are:

- Ethical review of provenance and sourcing of the material
- Legality of collection, transfer and use (national and international)

Each transfer of samples is undertaken according to a Research Collaboration Agreement or Material Transfer Agreement entered into by the Tree of Life collaborator, Genome Research Limited (operating as the Wellcome Sanger Institute) and in some circumstances other Tree of Life collaborators.

## Data Availability

European Nucleotide Archive: Panstrongylus geniculatus (kissing bug). Accession number PRJEB74974; https://identifiers.org/ena.embl/PRJEB74974. The genome sequence is released openly for reuse. The *Panstrongylus geniculatus* genome sequence is released openly for reuse. The assembly is provided by the Wellcome Sanger Institute Tree of Life Programme ( https://www.sanger.ac.uk/programme/tree-of-life/). All raw sequence data and the assembly have been deposited in INSDC databases. The genome will be annotated using available RNA-Seq data and presented through the Ensembl pipeline at the European Bioinformatics Institute. Raw data and assembly accession identifiers are reported in Table 1 and Table 2.

## Author information

Members of the Wellcome Sanger Institute Tree of Life Management, Samples and Laboratory team are listed here: https://doi.org/10.5281/zenodo.12162482.

Members of Wellcome Sanger Institute Scientific Operations: Sequencing Operations are listed here: https://doi.org/10.5281/zenodo.12165051.

Members of the Wellcome Sanger Institute Tree of Life Core Informatics team are listed here: https://doi.org/10.5281/zenodo.12160324.

Members of the Tree of Life Core Informatics collective are listed here: https://doi.org/10.5281/zenodo.12205391.
