# Supplementary material for: The genome sequence of the kissing bug, Panstrongylus geniculatus (Latreille, 1811)
Source: Wellcome Open Res. 2025 Feb 3;10:43. [Version 1] doi: 10.12688/wellcomeopenres.23631.1 (PMC11876800; doi:10.12688/wellcomeopenres.23631.1)
Supplement: Peer review history

# Reviewer report: Reviewer response for version 1

**DOI:** 10.21956/wellcomeopenres.26071.r118623

**Published:** 2025-03-03

**Authors:** Qianquan Chen

**Affiliations:** 1 Guizhou Normal University, Guiyang, Guizhou, China

*Panstrongylus geniculatus* (Latreille, 1811) is a significant vector of *Trypanosoma cruzi*, the etiological agent of Chagas disease (CD), which affects approximately 6–7 million people primarily in Latin America, with over 10,000 deaths annually. At the same time, adults of *P. geniculatus *exhibit sexual dimorphism. The genome sequence can contribute to research and management of Chagas disease.

Are sufficient details of methods and materials provided to allow replication by others?

Yes

Is the rationale for creating the dataset(s) clearly described?

Yes

Are the datasets clearly presented in a useable and accessible format?

Yes

Are the protocols appropriate and is the work technically sound?

Yes

Reviewer Expertise:

Eco-genomics

I confirm that I have read this submission and believe that I have an appropriate level of expertise to confirm that it is of an acceptable scientific standard.

# Reviewer report: Reviewer response for version 1

**DOI:** 10.21956/wellcomeopenres.26071.r118625

**Published:** 2025-02-24

**Authors:** Jerome H Hui

**Affiliations:** 1 The Chinese University of Hong Kong, Hong Kong, Hong Kong

In this data note, Canizales-Silva and colleagues reported the genome sequence of a female kissing or triatomine bug *Panstrongylus geniculatus *(Latreille, 1811)(Hemiptera, Reduviidae). Accordingly, it was Charles Darwin who first reported the triatomines in America. *P. geniculatus *is a potential vector of trypanosome *T* *rypanosoma cruzi*, a parasitic protozoan that is the causative agent of Chagas disease or American trypanosomiasis. According to the World Health Organisation, about 6-7 million people worldwide are estimated to be infected with *T. cruzi*. 

 Prior to this study, there are limited molecular data available for this species deposited on the NCBI database. This genome resource is thus important and will be very useful for further studies, such as understanding the biology and ecology of this disease vector, its sexual dimorphism, prevention of the transmission of diseases, as well as evolutionary and genomics questions related to insects more widely.

 This genome resource is excellent according to the summary statistics, with high BUSCO number scores, high sequence continuity (scaffold N50), and majority of sequences contained on the 10 pseudochromosomes (plus 2 sex chromosomes and mitochondrion). All in all, this is a valuable contribution.

Are sufficient details of methods and materials provided to allow replication by others?

Yes

Is the rationale for creating the dataset(s) clearly described?

Yes

Are the datasets clearly presented in a useable and accessible format?

Yes

Are the protocols appropriate and is the work technically sound?

Yes

Reviewer Expertise:

Genomics, evolution, invertebrates

I confirm that I have read this submission and believe that I have an appropriate level of expertise to confirm that it is of an acceptable scientific standard.
